# Bayesian adaptive designs for the optimisation of implementation strategies: a virtual re-execution of the Physically Active Children in Education (PACE) trials

**DOI:** 10.1186/s43058-026-01000-2

**Published:** 2026-06-22

**Authors:** Erin Nolan, Elizabeth Holliday, Christopher Oldmeadow, Alix Hall, Lucy Leigh, Nicole Nathan, Daniel Barker

**Affiliations:** 1 School of Medicine and Public Health, The University of Newcastle, Callaghan, NSW Australia; 2 Hunter Medical Research Institute, New Lambton Heights, NSW Australia; 3 National Centre of Implementation Science, The University of Newcastle, Newcastle, NSW Australia; 4 Hunter New England Population Health, Hunter New England Area Health Service, Newcastle, NSW Australia

**Keywords:** Optimisation, Bayesian, Adaptive design, Implementation, Trial re-execution

## Abstract

**Background:**

Current designs used to optimise implementation strategies (e.g., sequential cluster randomised controlled trials (cRCTs) and multi-arm cRCTs) are inefficient and resource intensive. Bayesian adaptive designs may offer a more efficient alternative.

**Methods:**

We conducted a virtual trial re-execution to assess the impact of using a Bayesian adaptive design for optimising an existing implementation strategy (Physically Active Children in education (PACE)) used to support the delivery of school-based physical activity. The two previous, sequential, two-arm cRCTs used to optimise PACE were combined into a single three-arm cRCT. We assessed the performance of a fixed version of this trial design compared to an adaptive version incorporating one, two, or three interim analyses in a virtual re-execution. Adaptions included arm dropping and early stopping for futility, noninferiority, or efficacy.

**Results:**

All adaptive designs stopped early for noninferiority, declaring the lower cost treatment as the optimal arm at interim analysis one. This was the same conclusion obtained in the fixed version of the three-arm cRCT and the original sequential two-arm cRCTs. Using adaptive designs, the same conclusion was reached using 50–75% fewer clusters. The adaptive trials would have taken approximately 40% less time than the sequential two-arm cRCTs, not accounting for the time to analyse the interim analysis. However, the treatment effect was biased towards the lower cost treatment, and this bias (away from the full-sample posterior estimate) increased when fewer clusters were randomised. The first 18 schools randomised were Catholic schools, and they responded better to the lower cost treatment compared to government schools. When the distribution of school type was balanced at each interim (i.e. matched the final sample proportions) the bias towards the lower cost treatment was reduced.

**Conclusions:**

Bayesian adaptive designs offer improved efficiency for trials aiming to optimise implementation strategies, reducing the time and sample size needed to find the optimal strategy. However, care is required to ensure confounding demographics are balanced at each interim analysis to reduce the risk of making a type 1 or 2 error or an incorrect adaptive design decision (e.g. dropping an effective arm, incorrectly stopping early).

**Clinical trial number:**

Not applicable.

**Supplementary Information:**

The online version contains supplementary material available at https://doi.org/10.1186/s43058-026-01000-2.

Contributions to the literature

- Current designs for optimising implementation strategies are often inefficient and resource intensive.
- We introduce Bayesian adaptive designs as a method that has the potential to reduce the time, cost, and clusters needed for studies aiming to optimise implementation strategies.
- This is one of the first papers to demonstrate the applicability of Bayesian adaptive designs for multi-arm, clustered trials in implementation science.
- We identify limitations to the use of Bayesian adaptive designs, namely the potential bias introduced if confounders aren’t balanced at interim analyses.

## Background

Optimisation is the process of identifying the best components or combination of components for an intervention or implementation strategy while managing real-world constraints (e.g. cost) [1]. In implementation science, accounting for real-world constraints could help prevent the attenuation of strategies once implemented. Implementation science, which focuses on translating evidence-based interventions into standard practice, has used various approaches for optimisation. Previously, trials have attempted to optimise using sequential two-arm cluster RCTs [2], but this process can be inefficient for the following reasons: the trial can perform only one comparison at a time, and the trial needs to be completed before another comparison can occur. Factorial designs have been recommended for optimisation [3] but have been considered prohibitively complex to use in this area [2]. Multi-arm trials (trials with more than two arms) offer a balanced approach to optimisation by allowing multiple components to be compared within a single trial, while being often less complex than factorial designs due to comparing fewer combinations of components. However, this lower complexity comes with the drawback that not every combination of components, or their interactions, can be evaluated.

Despite the advantages that multi-arm trials offer to optimising implementation strategies, these designs can still be inefficient because all arms receive an allotment of participants irrespective of performance, even when an arm may be shown ineffective if the data were analysed at an interim. Adaptive designs may offer a potential solution to these inefficiencies. The term adaptive refers to a priori modifications occurring to pre-specified design features during the conduct of the trial [4]. Adaptive designs can improve trial efficiency by enabling modifications to the trial to occur based on accumulated data, such as dropping ineffective arms to increase power to more promising arms or ending the trial early for efficacy, thus reducing the sample size and time needed to reach a conclusion. Adaptive designs have grown in popularity, due to the increased efficiency and flexibility they offer [5].

Alongside adaptive designs, Bayesian statistical methods can offer benefits over the more commonly used statistical approach of frequentist methods. Unlike frequentist methods that may have less clear inferential interpretations [6] Bayesian methods can readily incorporate accumulating data like that which is seen in adaptive designs [7, 8]. Bayesian models can also more readily converge compared to their frequentist counterparts when there is a low number of clusters [9]. Bayesian adaptive designs have been successfully used previously for clinical cluster randomised controlled trials (cRCTs), which is the most common research design used to evaluate the effect of implementation strategies [10–12]. A simulation by Harari et al. [12] found that using arm dropping in a three-arm cRCT decreased the proportion of clusters randomised into an ineffective arm from 33% to 20.9%. Despite their advantages, the use of Bayesian adaptive designs for optimising implementing strategies has not been explored.

We will explore how Bayesian adaptive designs could provide benefit to a trial aiming to optimise an implementation strategy (physical activity (PA) for school students) via a trial re-execution. Trial re-executions have been used previously to assess how Bayesian adaptive designs would apply to a real clinical trial [8, 13–15]. By applying Bayesian adaptive methods to a real-world example, trial re-executions provide evidence of these methods’ validity before resources are expended into a full trial. We apply a virtual re-execution to an implementation optimisation trial.

## Aim

Examine how Bayesian adaptive designs may impact the efficiency and conclusions of an implementation optimisation cRCT.

## Methods

### Case study

The Physically Active Children in Education (PACE) trials were a series of phase IV cluster randomised implementation trials that aimed to optimise an implementation strategy that supports classroom teachers’ delivery of a mandatory policy that requires them to schedule a total of 150 min of classroom physical activity per week [16–18]. The primary outcome was the weekly minutes of structured physical activity implemented by classroom teachers at 12 months. The first PACE trial was a pilot [16], the second PACE trial used a superiority framework to assess if the full PACE strategy was better than control [17], and the third PACE trial used a noninferiority framework to assess if a less expensive version of the PACE strategy was noninferior to the full PACE strategy [18]. Noninferiority trials assess whether a treatment or implementation strategy is not ‘substantially’ worse than another (often more expensive or intensive) treatment [19]. What is considered substantial is defined by the noninferiority margin.

The two non-pilot PACE trials are of interest for this paper. The first non-pilot PACE trial [17] will be referred to as PACE-S, and the second non-pilot PACE trial [18] will be referred to as PACE-NI. The full PACE strategy will be referred to as HD (for high dose) and the less expensive version of the PACE strategy will be referred to as LD (for low dose). The PACE-S and PACE-NI trials ran consecutively from October 2017 – December 2019. Detailed information about the PACE trials are presented elsewhere [17, 18].

### Simulation settings

The control arm from the PACE-S trial, the HD arms from the PACE-S and PACE-NI trials, and the LD arm from the PACE-NI trial were combined to form a three-arm adaptive cRCT. HD was considered the optimal treatment if it was superior to control and LD. LD was considered the optimal treatment if it was superior to control and noninferior (NI) to HD. The same noninferiority margin (-16.4 min) as in the PACE-NI trial was used.

The adaptive trial features considered were arm dropping, and stopping early for efficacy, futility, or noninferiority. If an arm was dropped from the trial, then the remaining clusters were randomised equally to the other arms. Only one arm could be dropped from the trial at a time.

**Fig. 1:**
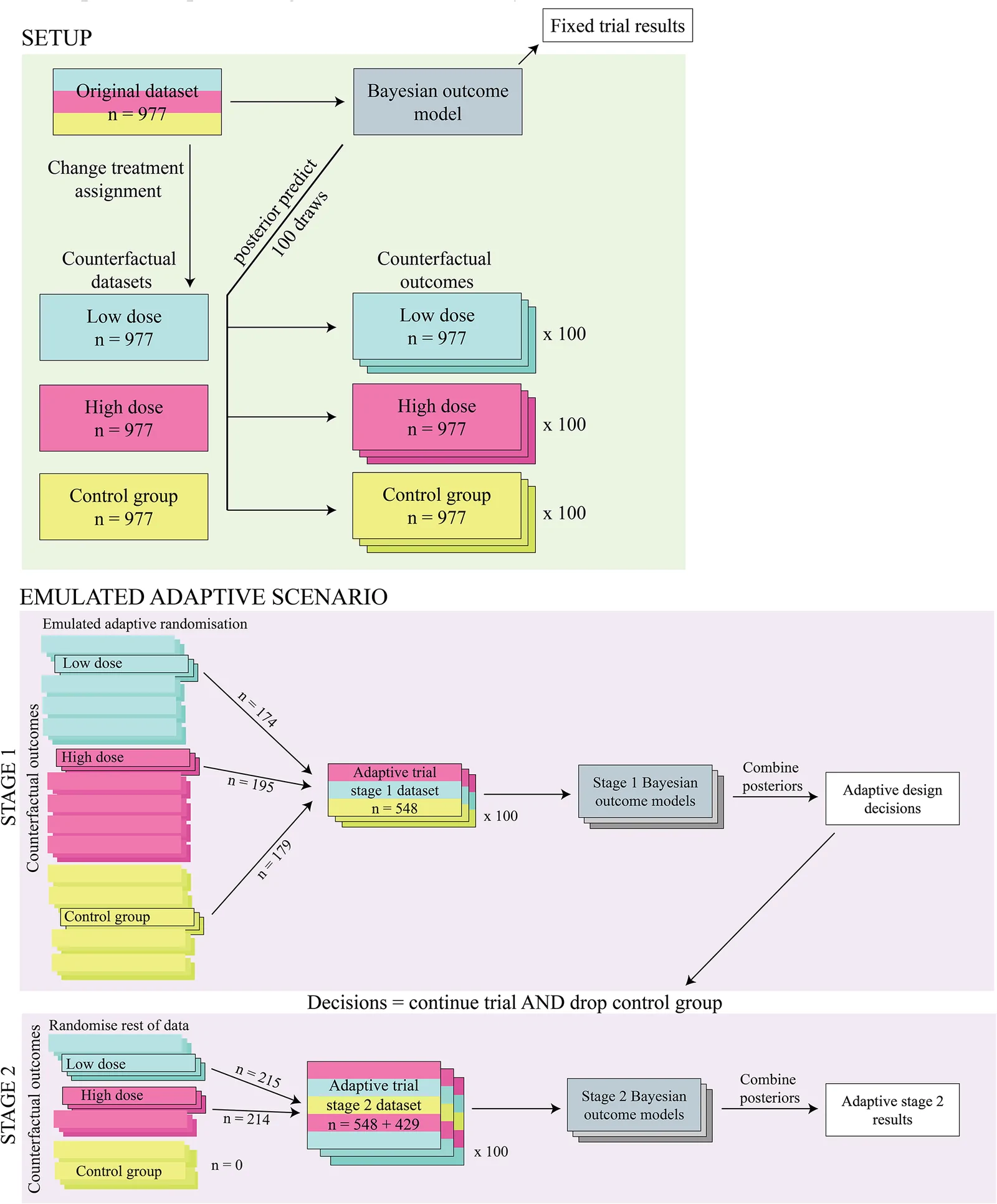
Flow diagram of method using example of adaptive design with 1 interim analysis. In the setup, the original dataset is taken to run the Bayesian outcome model. Three copies of that dataset are also made, where in each copy the participant has their treatment allocation changed to one of the treatments (so that every participant has the same allocation). Using the Bayesian outcome model and the three counterfactual datasets, 100 counterfactual outcomes are predicted for each participant. In the emulated adaptive scenario, the adaptive randomisation allocates participants to one of the three groups. For each participant, 100 counterfactual outcomes corresponding to the group they were assigned to are generated, producing 100 adaptive trial stage 1 datasets. Each dataset is then modelled, and the posteriors are combined to make an adaptive design decision. In this example, the decision is to continue the trial and drop the control group. In stage 2, the remaining participants are randomised and their respective counterfactual outcomes for that treatment group are added to the adaptive trial dataset. These datasets are then modelled and the posteriors are combined for the final trial results

Schools were randomised in the original order from the trials. Consistent with the original trials the schools’ remoteness (Major cities or Regional / remote) and school type (government, Catholic, or independent) were used as stratification variables with blocks of the same size. The model used is detailed below in the “Analysis” section. All data from the trials were used to model the outcome for the fixed trial. From the full model, the outcome for each teacher if their school had been randomised to a different arm (the counterfactual outcome) was sampled from the posterior predictive distribution. An example of the method used is shown in Fig. 1. One hundred counterfactual predictions per teacher ID were generated. Testing results with different seeds yielded minimal differences, and therefore 100 counterfactual predictions were deemed sufficient. The counterfactual predictions were set to missing if the outcome for that teacher ID was originally missing in the data. The predictions were then pooled and modelled (in the same model) in R using the brms package [20]. Bootstrapping / simulations were not used due to the clustered data and the presence of potential confounders, as this could have led to biased effects away from the original sample’s effects if these factors were ignored. Furthermore, there were too few teachers within the cluster with the same confounding demographics and baseline characteristics, limiting the ability to resample.

### Analysis

Bayesian hierarchical models were used to compare the minutes of scheduled weekly structured physical activity (PA) between HD, LD, and control. A normal distribution was used, with a random intercept for school to account for clustering and fixed effects for treatment arm (HD / LD / control), baseline PA, socio-economic status (SES) (most disadvantaged / least disadvantaged), remoteness (major cities / regional or remote), school type (Catholic / government / independent), and trial (PACE-S / PACE-NI). For SES and remoteness, the schools’ postcodes were used to obtain the corresponding Australian Bureau of Statistics 2016 classification for socio-economic index [21] and remoteness structures [22], respectively.

Vague priors ($\:N\left(\mathrm{0,1000}\right))$ were used for the intercept ($\:{\beta\:}_{0}$) and fixed effects ($\:{\beta\:}_{1},\dots\:,\:{\beta\:}_{6})$. For the random intercept by school ID ($\:{\alpha\:}_{k})$ a normal distribution was used ($\:\mathrm{N}(0,{\upsigma\:}))$, where the standard deviation $\:\sigma\:$ assumed a prior of: $\:\sigma\:\sim Half{T}_{3}\left(\mathrm{0,62}\right)$, where 0 and 62 were the default option in brms, reflecting the standard deviation of the outcome in the full dataset. The full model was as follows:

1 $$\begin{aligned}\:{Y}_{ik}&=\:{\beta\:}_{0}+{\beta\:}_{1}{X}_{1ik}+{\beta\:}_{2}{X}_{2ik}+{\beta\:}_{3}{X}_{3ik}+{\beta\:}_{4}{X}_{4ik}\cr&\quad+{\beta\:}_{5}{X}_{5ik}+\:{\beta\:}_{6}{X}_{6ik}+{\alpha\:}_{k}+{\epsilon}_{ik}\end{aligned}$$

Where Y is the PA, * i* is the participant, * k* is the cluster, $\:{X}_{1}$ is the treatment arm, $\:{X}_{2}$ is the baseline PA, $\:{X}_{3}$ is socio-economic status, $\:{X}_{4}$ is remoteness, $\:{X}_{5}$ is the trial, X_6_ is the school type, and *ε* is the individual-level error. The analysis was conducted in R via the brms package [20, 23], which uses Stan [24]. Draws from the posterior distribution were obtained using the No-U-Turn-Sampler [24]. Two-thousand five-hundred warm-ups and 2,500 draws were used with four chains, totalling 10,000 warm-ups and 10,000 draws. Missing follow-up PA values were imputed using full Bayesian imputation within the model, assuming the data were missing at random (MAR). Posterior checks were performed by running trace plots and assessing the $\:\widehat{R}$ and effective sample size in the bulk and tail. The variance partition coefficients (VPC) for the PACE-S and PACE-NI trials, and the trials combined were calculated. The VPC quantifies the correlation of response values from participants within a cluster, and is equivalent to the intra-class correlation [25].

### Adaptions

We chose potential adaptations in consultation with original researchers of the trial. Designs offering potentially increased efficacy and deemed practically and statistically feasible were included. The selected adaptations included arm dropping and stopping early for futility, efficacy, or noninferiority. The trials were run with either zero (fixed), one, two, or three interim analyses. The designs used and number and timing of interim analyses are detailed in Table 1.

**Table 1:** Designs considered for the re-execution of the PACE trials, along with the number and timing of the interim analyses (rounded to the nearest cluster)

Design	*N* interims	Timing of interim analysis
Fixed	Zero	NA
Arm dropping and stopping early for futility / efficacy / noninferiority	One	1/2 clusters with outcome
	Two	Every 1/3 clusters with outcome
	Three	Every 1/4 clusters with outcome

For the rules to determine arm dropping at the interim analyses, the cut point of 0.9 was used as it represented a high posterior probability and was used in previous trial re-executions [18]. The control arm would be dropped if the posterior probability that the high dose arm and low dose arm had greater treatment effects than the control arm was > 0.9:

$$\:P\left(HD>Ctrl\right)>0.9\:\ \&\ \:P\left(LD>Ctrl\right)>0.9$$

The high dose arm would be dropped if the posterior probability that the low dose and control arms had a larger treatment effect than the high dose arm was > 0.9:

$$\:P\left(LD>HD\right)>0.9\:\ \&\ \:P\left(Ctrl>HD\right)>0.9$$

The low dose arm would be dropped if the posterior probability that the low dose arm was inferior to the high dose arm and had a smaller treatment effect than the control arm was > 0.9:

$$\:P\left(HD-16.4>LD\right)>0.9\:\ \&\ \:P\left(Ctrl>LD\right)>0.9$$

The trial could stop early for efficacy, noninferiority, or futility. To determine the cut-points for early stopping, we simulated the trial using the expected trial characteristics used to calculate power in the PACE-NI trial, with the addition of a third arm. For the early stopping adaptions, we chose a cut-point of 0.95 as in simulation it corresponded to a maximum type 1 error rate of 5%, that is, the trial would declare success (noninferiority or superiority) 5% of the time when the strategies had no true effect. Simulation methods and results are detailed in Supplementary File 1. The rules to determine early stopping were as follows. If the posterior probability that the high dose arm was superior to the control and low dose arms was > 0.95, then the trial would stop early for superiority:

$$\:P\left(HD>Ctrl\right)>0.95\:\ \&\ \:P\left(HD>LD\right)>0.95$$

If the posterior probability that the low dose arm was noninferior (NI) to the high dose arm and superior to the control arm was > 0.95, then the trial would stop early for noninferiority:

$$\:P\left(LD>\:HD-16.4\right)>0.95\:\ \&\ \:P\left(LD>Ctrl\right)>0.95$$

If both the superiority and noninferiority conditions were met, then the trial would stop early for noninferiority. If the posterior probability that the low and high dose arms were superior to control was < 0.1, then the trial would stop early for futility:

$$\:P\left(HD>Ctrl\right)<0.1\:\ \&\ \:P\left(LD>Ctrl\right)<0.1$$

### Sensitivity analysis

Since the order of randomisation meant that school type was unbalanced early in the trial, a sensitivity analysis was performed. First, to determine if there was a differential effect of the treatment by school type, the same model described in Eq. 1 was run, but with an interaction effect between the treatment arm ($\:{X}_{1}$) school type (X_6_) added.

The trial was then rerun with a balanced school type at each interim analysis. That is, at each stage the proportion of each school type randomised was approximately equal to the final overall proportion. The order of randomisation was maintained within each school type. No other changes to the randomisation order or analysis were made.

To assess whether the primary results were robust against violations of the MAR assumption, a sensitivity analysis was run using a delta-adjustment [26]. The same models for the primary analyses were used, but an offset of -180 min/week was also applied to the predicted values for the missing follow-up PA observations.

## Results

Across both trials 109 schools and 977 teachers were randomised. 

**Table 2:** School level demographics of analysed schools in the PACE-S and PACE-NI trials. Differences in the distribution were assessed by a chi-sq p-value

Demographics	PACE-S	PACE-NI	Chi-sq *p*-value
**School type**			0.003^1^
Catholic	21 (34%)	5 (10%)	
Government	40 (66%)	41 (85%)	
Independent	0 (0%)	2 (4.2%)	
**Socioeconomic status**			0.2
Most disadvantaged	38 (62%)	36 (75%)	
Least disadvantaged	23 (38%)	12 (25%)	
**Remoteness**			0.11
Major Cities	36 (59%)	21 (44%)	
Regional/Remote	25 (41%)	27 (56%)	

^**1**^Fisher’s exact

The PACE-S trial randomised 21 / 60 (34%) Catholic schools, whereas the PACE-NI trial randomised 5 / 48 (10%) Catholic schools. The distribution of school type was different between trials (*p* = 0.003) (Table 2). There was little evidence that the remaining school demographics were unbalanced (*p* > 0.05). There were on average 8.96 teachers per school, with a range of one to 31 teachers per school. The VPCs of the PACE-S and PACE-NI trials were 0.08 and 0.06, respectively. The VPC of the two trials combined was 0.06. For the primary outcome, 390 / 977 (40%) responses were missing. 

**Table 3:** Adaptive design decisions, conclusions, and mean difference of weekly PA time administered by teachers by trial design

*N* interims			Posterior probabilities				Mean difference (95% CrI)	
	Adaptive design decisions	Conclusion	*P*(LD > HD-16.4)	*P*(LD > HD)	*P*(LD> Ctrl)	*P*(HD> Ctrl)	LD - HD	LD - Ctrl
**Fixed (0)**	NA	LD optimal	0.98	0.47	> 0.99	> 0.99	-0.5 (-15.7, 14.4)	46.9 (28.2, 65.7)
*Interim 1*								
One	Stop early for NI	LD optimal	0.95	0.60	> 0.99	> 0.99	3.1 (-18.6, 27.7)	53.8 (30.1, 79.4)
Two	Stop early for NI	LD optimal	> 0.99	0.84	> 0.99	> 0.99	12.7 (-11.9, 40.3)	55.5 (30.8, 87.0)
Three	Stop early for NI	LD optimal	> 0.99	0.87	> 0.99	> 0.99	15.7 (-9.11, 44.1)	64.3 (37.5, 93.6)

**Table 4:** Number of schools and teachers randomised by design

*N* interims			Schools assigned to arm (*n* %)			Teachers assigned to arm (*n* %)		
	*N* schools	*N* teachers	Control	LD	HD	Control	LD	HD
Fixed (0)	109	977	30 (28%)	24 (22%)	55 (50%)	282 (29%)	207 (21%)	488 (50%)
One	55	548	18 (33%)	17 (31%)	20 (36%)	179 (33%)	174 (32%)	195 (36%)
Two	36	389	12 (33%)	11 (31%)	13 (36%)	128 (33%)	117 (30%)	144 (37%)
Three	27	282	8 (30%)	9 (33%)	10 (37%)	86 (30%)	91 (32%)	105 (37%)

When the two PACE trials were combined into a three-arm fixed cRCT, the trial concluded that LD is noninferior to HD and superior to control and thus the optimal treatment (P(LD > HD-16.4) = 0.98, P(LD > Ctrl) > 0.99) (Table 3). Teachers in the LD schools implemented on average 0.5 less minutes of PA than teachers in HD schools, and 46.9 min more than teachers in Ctrl schools.

This trial used the entire sample of 109 schools and 977 teachers (Table 4). When the trial was executed using adaptive designs, the one, two, and three interim designs all stopped early for noninferiority at interim one and concluded LD was the optimal arm. The one, two, and three interim trials randomised 55, 36, and 27 schools before reaching a trial conclusion, respectively. The fixed trial randomised 30 (28%) schools to Control, 24 (22%) to LD, and 55 (50%) to HD. This trial randomised 282 (29%) teachers to control, 207 (21%) to LD, and 488 (50%) to HD. In the one interim adaptive trial, 10 (33%) schools were randomised to Control, 17 (31%) to LD, and 20 (36%) to HD. In the two interim adaptive trial, 12 (33%) schools were randomised to Control, 11 (31%) to LD, and 13 (36%) to HD. In the three interim adaptive trial, 8 (30%) schools were randomised to Control, 9 (33%) to LD, and 10 (37%) to HD.

### Sensitivity analysis

The first 18 schools randomised in the trial were Catholic schools, and they responded more favourably to LD than HD compared to the government schools. In the full model with the added interaction effect, the mean difference in PA minutes implemented between LD and HD was 16.73 min (95% CrI − 12.91, 45.87) in Catholic schools, -8.43 min (95% CrI − 24.33, 7.60) in government schools, and 61.98 min (95% CrI 3.88, 121.50) in independent schools. 

**Table 5:** Balanced randomisation order for school type (~24% Catholic ~74% government ~2% independent). Adaptive design decisions, conclusions, and mean difference of weekly PA time administered by teachers by trial design

*N* interims			Posterior probabilities				Mean difference (95% CrI)		
	Adaptive design decisions	Conclusion	*P*(LD > HD-16.4)	*P*(LD > HD)	*P*(LD> Ctrl)	*P*(HD> Ctrl)	LD - HD	LD - Ctrl	*N* schools
*Interim 1*									
One	Drop control		0.94	0.57	> 0.99	> 0.99	2.0 (-20.7, 26.7)	52.0 (27.6, 78.8)	55
Two	Stop early for NI	LD optimal	0.96	0.66	> 0.99	> 0.99	5.6 (-19.6, 34.1)	50.1 (21.6, 81.4)	36
Three	Stop early for NI	LD optimal	0.95	0.68	> 0.99	> 0.99	7.0 (-21.0, 35.1)	50.7 (18.7, 85.9)	27
*Trial end*									
One	NA	LD optimal	0.98	0.54	> 0.99	> 0.99	1.0 (-16.5, 19.1)	51.5 (27.9, 75.9)	109

When the trial had a balanced randomisation order, that is, the proportion of school types was roughly equal to the final sample, the one interim adaptive trial dropped the control arm at interim one and concluded LD was the optimal arm at the full sample size (P(LD > HD-16.4) = 0.98, P(LD > Ctrl) > 0.99) (Table 5). The two and three interim trials both stopped early for noninferiority at interim one, concluding LD was the optimal arm (two interim trial: P(LD > HD-16.4) = 0.96, three interim trial: P(LD > HD-16.4) = 0.95). The same number of schools as in the primary analysis were randomised in the sensitivity analysis except for the one interim design, which randomised all 109 schools instead of 55.

Within trial, the percent of missing follow-up PA values was within 2% between treatment groups. The missingness was different between trials however, with 35% missing in PACE-S and 48% missing in PACE-NI. The trial conclusions remained consistent when assuming missing follow-up PA values were 180 min/week lower than non-missing values (Supplementary File 2).

## Discussion

We conducted a re-execution of the PACE trials to examine the effect of Bayesian adaptive designs, particularly arm dropping and early stopping, on implementation optimisation trials.

The fixed trials, when combined, concluded that LD was the optimal treatment. This conclusion was consistent when the trials were run with an adaptive design. Using the original randomisation order, the designs with one, two, and three interim analyses all stopped early for noninferiority at the first interim and declared LD as the optimal treatment. The adaptive designs reached the same conclusion as the fixed trial but with 50–75% less clusters randomised. In addition, treatment arms were more balanced, with ~ 36% of clusters randomised to the HD arm instead of 50% in the fixed trial. The adaptive trials were more efficient than the fixed trial. The PACE-S and PACE-NI trials ran over two years and two months. The first 55 schools were randomised from October 2017 to February 2018. With the 12-month outcome included the adaptive trial with one interim analysis would have taken approximately 16 months to reach a conclusion, without accounting for the time to perform the interim analysis. This is approximately a 40% reduction in the trial length to reach the same conclusion. Running all arms consecutively also improved time efficiency. Under a fixed 3‑arm design, the trial would take 16 to 20 months, depending on the speed of recruitment. If all schools were recruited and baseline outcomes measured within the same 4‑month period, the fixed 3‑arm trial could reach completion in the same time as the adaptive trial. However, if recruitment and baseline measurement progressed at the same rate as observed (taking 8 months), the adaptive trial would be approximately 20% faster. An additional benefit of the multi-arm trial is that it could conclude that LD was superior to control, which had not been established until now.

There was evidence of bias (away from the full-sample posterior estimate) arising from the original randomisation order, with the estimated effect size for LD being larger when fewer clusters were randomised. This was partly explained by differences in response by school type: Catholic and independent schools responded more favourably to LD than government schools, and the first 18 schools randomised were Catholic, leading interim analyses conducted after fewer clusters to be biased towards favouring LD. Although the two and three interim adaptive trials still stopped early for noninferiority at the first interim, balancing school type in the randomisation reduced (but did not remove) the trend favouring LD when fewer schools were randomised.

This residual effect may reflect differences between trials, with schools in the PACE NI trial responding more favourably to the HD strategy than those in PACE S, as well as temporal trends or improvements in implementation over time. This shift highlights a broader limitation of sequential RCTs: treatments introduced later may not be directly comparable with earlier ones due to time dependent changes. Our use of ‘trial’ as a fixed effect to account for differences between trial could also account for differences in time. In a real adaptive trial though, ‘trial’ wouldn’t be used as there would not be different trials to account for. Instead, to account for temporal trends it may be useful to include the trial stage as a fixed effect if temporal trends are anticipated. There are also more complex methods to account for temporal trends, such as spline regression [27] the Bayesian Time Machine method [28], but the method to choose depends on the trial design and context.

While the order of randomisation is generally unimportant in fixed trials (since outcomes are analysed only at completion) it is particularly important in adaptive designs. If the order of randomisation is unbalanced, interim decisions in adaptive trials may be biased in both individually and cluster randomised designs. This risk increases when treatment effects interact with participant or cluster characteristics and may be intensified in cluster trials due to simultaneous individual and cluster level interactions.

There are several methods for improving balance at each interim including stratified randomisation, dynamic block randomisation, and covariate adaptive randomisation [29, 30]. Stratified randomisation is most effective when there are few confounder levels and cluster enrolment is known in advance, thus avoiding potentially very small or empty strata [29]. When the number of confounder levels becomes large, or the enrolment is unpredictable, methods like dynamic block randomisation or covariate adaptive randomisation may be more suitable. In all cases however, potential confounders must be identified in advance to enable the balancing across groups.

In this trial, there was a relatively low VPC of 0.06, multiple school level demographics and a wide range of the number of teachers per school (one to 31). The adaptive trials may have performed particularly well despite demographic and cluster size imbalance partially because of the low VPC. If the VPC had been larger, then the adaptive design decisions would have been more likely to be incorrect.

This study was novel in that it was one of the first times a noninferiority conclusion was added into the early stopping design in a Bayesian multi-arm cRCT. A noninferiority rule was previously used in the EXAMINE trial [31] but this was under a frequentist framework. This is likely due to previous literature advising against the use of stopping early for noninferiority [32–34]. While previous literature has advised against this practice, the reasons for this do not necessarily apply to this study. The reasons include:

1. The subjective nature of the noninferiority margin.
2. It being considered less ethical need to stop early for noninferiority when the comparison group is still receiving a treatment.
3. The increased cost and time needed to conduct interim analyses.

However, these concerns do not necessarily apply to noninferiority in this context. First, the argument that stopping for noninferiority shouldn’t occur as the noninferiority margin is subjective [34] does not directly apply to this study as we still required that the less expensive treatment was better than control – i.e. there is only potential for partial subjectiveness. If a trial seeks to include designs that allow for stopping for noninferiority, then the inclusion of a control group where superiority must also be established can help reduce the weakness of the noninferiority conclusion. Second, in this study, there is a non-active control group so it still may be more ethically sound to stop early for noninferiority to allow for more participants to receive treatment. However, it is often less ethically pressing to not receive treatment in implementation trials (but not always) since the implementation strategies do not have a direct effect on a patient’s/client’s health outcome [35]. Third, when also running interim analyses for other adaptive decisions (early stopping for futility / efficacy, and arm dropping), the inclusion of early stopping for noninferiority adds minimal increased cost/time.

The usefulness of the adaptive designs would be increased if the time it took to obtain the outcome was shorter. Even though they are more efficient for this trial and other sequential two-arm cRCTs, the benefits of adaptive designs aren’t fully realised when decisions are made from an outcome at 12 months. Using an interim outcome, for example PA collected at 6 months, could be useful but the correlation between the interim outcome and the primary outcome needs to be understood before its use. For the intermediate outcome to be considered valid for use it needs to have a high correlation to the primary outcome [36]. If it is not highly correlated to the primary outcome, then there is an increased risk for incorrect adaptive design decisions. The increased burden of collecting the intermediate outcome on the participants and researchers also needs to be evaluated. The downsides of the burden, such as increased researcher cost or participant dropout, might outweigh the benefits of gathering the outcome sooner. How the characteristics of the clusters affect the value of the surrogate outcome has not been explored.

In addition to the long time required to collect the outcome for adaptive design decisions, the outcome was also missing for approximately 40% of participants. Despite this, the adaptive designs still provided efficiency gains. In addition, the results were robust against missing not at random assumptions, with conclusions remaining consistent even when an extreme departure from MAR (-180 min/week) was tested. A high rate of missingness in the outcome used to make adaptive design decisions can bias interim effect estimates away from their true values, both impacting the risk of incorrectly stopping and incorrectly dropping (or not dropping) an arm [37]. If the outcome is missing at random then the missingness may introduce less bias. Part of the missingness in the PACE trials could be missing at random, with some missing data being attributed to staff turnover, staff not being available during the collection period, and the accessible practice for teachers to transfer between schools [38].

There are some practical aspects of running the PACE trials with adaptive designs that need to be considered. The trials had originally randomised groups that had the ethics approval accepted first. If the trial was run with adaptive designs, the start of the trial would need to have been delayed to ensure a more balanced group of schools were ready for randomisation. However, it cannot be determined which demographics or characteristics will confound the outcome. Researchers may need explore previous literature to determine which demographics and characteristics may be confounders and balance on as many as possible. A further practical consideration pertains to funding and ethics approval to run adaptive designs. Historically it was more difficult to apply for grants or get ethics approval for adaptive designs [4] but since the influx of adaptive designs to assess treatments for covid, the process is less difficult. It is recommended to show the best and worst-case scenarios in terms of sample sizes / cost or present a realistic range of values to a funding body [39].

### Limitations

We did not examine how adaptive designs influenced the ability to measure secondary outcomes. These secondary outcomes would likely be negatively impacted if the trial stopped early, in that they would have less power.

Because the randomisation was different between the trials, and because we did not obtain local health district, we could not perfectly replicate the randomisation scheme. This may mean that the results would be slightly different to what could have happened if the adaptive design was used. In addition, we did not perfectly replicate the models from the trials. The models were different between the PACE-S and PACE-NI trials. The PACE-S trial was in a frequentist framework, and while the PACE-NI trial was conducted in a Bayesian framework, it did not include potential cluster level confounders. The model we described in methods was also used for a fixed version of the combined trials, enabling us to compare the outcomes between the fixed and adaptive designs. However, differences in our results to the original trials may partially be due to the different modelling strategies. Where we adjusted for school demographics in a Bayesian framework, the PACE-S trial used a frequentist framework, and the PACE-NI trial did not adjust for school demographics. The different modelling strategies can result in different treatment effects estimated and different conclusions reached.

## Conclusion

Bayesian adaptive designs have the potential to greatly increase the efficiency of cRCT trials aiming to optimise implementation strategies. However, if confounders are not balanced at the interim analysis, then the results can be biased. The order of randomisation needs to be balanced. Through a real-world example, we demonstrated the potential benefits of Bayesian adaptive designs for implementation optimisation cRCTs.

## Supplementary Information

Below is the link to the electronic supplementary material.

Supplementary Material 1

Supplementary Material 2

## Data Availability

Request for data and other study documents should be made to the senior investigators of the PACE trials. The code for the trial re-execution is located at https://github.com/ErnKNolan/PACE_reex.

## References

[1] Wolfenden L, Bolsewicz K, Grady A, McCrabb S, Kingsland M, Wiggers J, et al. Optimisation: Defining and Exploring a Concept to Enhance the Impact of Public Health Initiatives. Health Res Policy Syst. 2019;17(1):108. 10.1186/s12961-019-0502-6. 31888666 PMC6937822

[2] Lane C, Nathan N, Wiggers J, Hall A, Shoesmith A, Bauman A, et al. Learning Health System to Rapidly Improve the Implementation of a School Physical Activity Policy. Implement Sci Commun. 2024;5(1):85. 10.1186/s43058-024-00619-3. 39085972 PMC11292924

[3] Collins L. Optimization of behavioral, biobehavioral, and biomedical interventions: the multiphase optimization strategy (MOST). Springer; 2018.

[4] Pallmann P, Bedding AW, Choodari-Oskooei B, Dimairo M, Flight L, Hampson LV, et al. Adaptive designs in clinical trials: why use them, and how to run and report them. BMC Med. 2018;16(1):29. 10.1186/s12916-018-1017-7. 29490655 PMC5830330

[5] Bothwell LE, Avorn J, Khan NF, Kesselheim AS. Adaptive Design Clinical Trials: A Review of the Literature and Clinicaltrials.gov. BMJ Open. 2018;8(2):e018320. 10.1136/bmjopen-2017-018320. 29440155 PMC5829673

[6] Berry SM, Carlin B, Lee JJ, Müller P. Bayesian Adaptive Methods for Clinical Trials. 1st ed. Boca Raton: Chapman & Hall/CRC; 2010. (Chapman & Hall/CRC biostatistics series; 38).

[7] Chow SC, Chang M. Adaptive Design Methods in Clinical Trials - a Review. Orphanet J Rare Dis. 2008;3:11. 10.1186/1750-1172-3-11 . PubMed Central PMCID: PMC2422839. 18454853 PMC2422839

[8] Ryan EG, Lamb SE, Williamson E, Gates S. Bayesian adaptive designs for multi-arm trials: an orthopaedic case study. Trials. 2020;21(1):83. 10.1186/s13063-019-4021-0 . PubMed Central PMCID: PMC6961269. 31937341 PMC6961269

[9] McNeish D. On Using Bayesian Methods to Address Small Sample Problems. Struct Equation Modeling: Multidisciplinary J. 2016;23(5):750–73. 10.1080/10705511.2016.1186549.

[10] Shen J, Golchi S, Moodie EE, Benrimoh D. Bayesian Group Sequential Designs for Cluster-Randomized Trials. Stat. 2022;11(1):e487. 10.1002/sta4.487.

[11] Hemming K, Martin J, Gallos I, Coomarasamy A, Middleton L. Interim Data Monitoring in Cluster Randomised Trials: Practical Issues and a Case Study. Clin Trails. 2021;18(5):552–61. 10.1177/17407745211024751. PMC8479148 34154426

[12] Harari O, Park JJH, Lat PK, Mills EJ. Adaptive Designs in Public Health: Vaccine and Cluster Randomized Trials Go Bayesian. Stat Med. 2024;43(14):2811–29. 10.1002/sim.10104. 38716764

[13] Ryan EG, Bruce J, Metcalfe AJ, Stallard N, Lamb SE, Viele K, et al. Using Bayesian Adaptive Designs to Improve Phase III Trials: A Respiratory Care Example. BMC Med Res Methodol. 2019;19(1):99. 10.1186/s12874-019-0739-3. 31088354 PMC6515675

[14] Wang Y, Travis J, Gajewski B. Bayesian Adaptive Design for Pediatric Clinical Trials Incorporating a Community of Prior Beliefs. BMC Med Res Methodol. 2022;22(1):118. 10.1186/s12874-022-01569-x. 35448963 PMC9027907

[15] Hong W, McLachlan SA, Moore M, Mahar RK. Improving Clinical Trials Using Bayesian Adaptive Designs: A Breast Cancer Example. BMC Med Res Methodol. 2022;22(1):133. 10.1186/s12874-022-01603-y. 35508968 PMC9066830

[16] Nathan NK, Sutherland RL, Hope K, McCarthy NJ, Pettett M, Elton B, et al. Implementation of a School Physical Activity Policy Improves Student Physical Activity Levels: Outcomes of a Cluster-Randomized Controlled Trial. J Phys Activity Health. 2020;17(10):1009–18. 10.1123/jpah.2019-0595. 32919383

[17] Nathan N, Hall A, McCarthy N, Sutherland R, Wiggers J, Bauman AE, et al. Multi-strategy intervention increases school implementation and maintenance of a mandatory physical activity policy: outcomes of a cluster randomised controlled trial. Br J Sports Med. 2022;56(7):385. 10.1136/bjsports-2020-103764. 34039583 PMC8938653

[18] Lane C, Wolfenden L, Hall A, Sutherland R, Naylor PJ, Oldmeadow C, et al. Optimising a multi-strategy implementation intervention to improve the delivery of a school physical activity policy at scale: findings from a randomised noninferiority trial. Int J Behav Nutr Phys Activity. 2022;19(1):106. 10.1186/s12966-022-01345-6. PMC9392334 35987776

[19] Hahn S. Understanding Noninferiority Trials. Korean J Pediatr. 2012;55(11):403–7. 10.3345/kjp.2012.55. .11.403 PubMed PMID: 23227058; PubMed Central PMCID: PMC3510268. 23227058 PMC3510268

[20] Bürkner PC. brms: An R Package for Bayesian Multilevel Models Using Stan. J Stat Softw. 2017;80(1):1–28. 10.18637/jss.v080.i01.

[21] Australian Bureau of Statistics. 2033.0.55.001 - Census of population and housing: socio-economic indexes for areas (SEIFA), Australia, 2016 [Internet]. Available from: https://www.abs.gov.au/ausstats/abs@.nsf/mf/2033.0.55.001.

[22] Australian Bureau of Statistics. 1270.0.55.005 - Australian Statistical Geography Standard (ASGS): Volume 5 - Remoteness Structure, July 2016 [Internet]. Available from: https://www.abs.gov.au/ausstats/abs@.nsf/mf/1270.0.55.005.

[23] R Development Core Team. R: A Language and Environment for Statistical Computing. Vienna, Austria: R Foundation for Statistical Computing; 2023.

[24] Stan Development Team. Stan Modeling Language Users Guide and Reference Manual. 2023.

[25] Merlo J, Wagner P, Leckie G. A Simple Multilevel Approach for Analysing Geographical Inequalities in Public Health Reports: The Case of Municipality Differences in Obesity. Health Place. 2019;58:102145. 10.1016/j.healthplace.2019.102145. 31195211

[26] Rubin DB. Formalizing Subjective Notions About the Effect of Nonrespondents in Sample Surveys. J Am Stat Assoc. 1977;72(359):538–43. 10.2307/2286214. Located at: JSTOR.

[27] Krotka P, Posch M, Gewily M, Höglinger G, Bofill Roig M. Statistical Modeling to Adjust for Time Trends in Adaptive Platform Trials Utilizing Non-Concurrent Controls. Biom J. 2025;67(3):e70059. 10.1002/bimj.70059 . PubMed PMID: 40491339; PubMed Central PMCID: PMC12150008. 40491339 PMC12150008

[28] Saville BR, Berry DA, Berry NS, Viele K, Berry SM. The Bayesian Time Machine: Accounting for temporal drift in multi-arm platform trials. Clin Trials. 2022;19(5):490–501. doi:10.1177/17407745221112013 PubMed PMID: 35993547. 35993547 10.1177/17407745221112013

[29] Lin Y, Zhu M, Su Z. The pursuit of balance: An overview of covariate-adaptive randomization techniques in clinical trials. Contemp Clin Trials. 2015;45:21–5. 10.1016/j.cct.2015.07.011. 26244705

[30] Xiao L, Lavori PW, Wilson SR, Ma J. Comparison of dynamic block randomization and minimization in randomized trials: a simulation study. Clin Trials. 2011;8(1):59–69. doi:10.1177/1740774510391683 PubMed PMID: 21335590; PubMed Central PMCID: PMC4296975. 21335590 10.1177/1740774510391683 PMC4296975

[31] White William B, Cannon Christopher P, Heller Simon R, Nissen Steven E, Bergenstal Richard M, Bakris George L, et al. Alogliptin after acute coronary syndrome in patients with type 2 diabetes. N Engl J Med. 369(14):1327–35. 10.1056/NEJMoa1305889. 23992602

[32] Piaggio G, Elbourne DR, Pocock SJ, Evans SJW, Altman DG. Reporting of Noninferiority and Equivalence Randomized Trials: Extension of the CONSORT 2010 Statement. JAMA. 2012;308(24):2594–604. 10.1001/jama.2012.87802. 23268518

[33] D’Agostino Sr RB, Massaro JM, Sullivan LM. Non-Inferiority Trials: Design Concepts and Issues – the Encounters of Academic Consultants in Statistics. Stat Med. 2003;22(2):169–86. 10.1002/sim.1425. 12520555

[34] Korn EL, Freidlin B. Interim Monitoring for Non-Inferiority Trials: Minimizing Patient Exposure to Inferior Therapies. Ann Oncol. 2018;29(3):573–7. 10.1093/annonc/mdx788. 29267937 PMC6248434

[35] Smith JD, Li DH, Merle JL, Keiser B, Mustanski B, Benbow ND. Adjunctive Interventions: Change Methods Directed at Recipients That Support Uptake and Use of Health Innovations. Implement Sci. 2024;19(1):10. 10.1186/s13012-024-01345-z. 38331832 PMC10854146

[36] Krendyukov A, Singhvi S, Zabransky M. Value of Adaptive Trials and Surrogate Endpoints for Clinical Decision-Making in Rare Cancers. Front Oncol. 2021;11:636561. 10.3389/fonc.2021.636561 . PubMed PMID: 33763370; PubMed Central PMCID: PMC7982798. 33763370 PMC7982798

[37] Lauffenburger JC, Choudhry NK, Russo M, Glynn RJ, Ventz S, Trippa L. Designing and Conducting Adaptive Trials to Evaluate Interventions in Health Services and Implementation Research: Practical Considerations. BMJ Med. 2022;1(1):e000158. 10.1136/bmjmed-2022-000158 . PubMed PMID: 36386444; PubMed Central PMCID: PMC9650931. 36386444 PMC9650931

[38] NSW Government - Education. Your career journey [Internet]. 2025 [cited 2025 Jun 14]. Available from: https://education.nsw.gov.au/about-us/careers-at-education/why-work-at-education/your-career-journey#Job1.

[39] Zhang Q, Dimairo M, Julious SA, Lewis J, Yu Z. Reporting and Communication of Sample Size Calculations in Adaptive Clinical Trials: A Review of Trial Protocols and Grant Applications. BMC Med Res Methodol. 2024;24(1):216. 10.1186/s12874-024-02339-7. 39333920 PMC11430544

