# Supplementary material for: Bayesian adaptive designs for the optimisation of implementation strategies: a virtual re-execution of the Physically Active Children in Education (PACE) trials
Source: Implement Sci Commun. 2026 Jun 22;7:179. doi: 10.1186/s43058-026-01000-2 (PMC13548693; doi:10.1186/s43058-026-01000-2)
Supplement: Supplementary file 1 [file 43058_2026_1000_MOESM1_ESM.docx]

*Supplementary file 1: Simulation settings and results to determine cut-points for noninferiority and superiority margins.*

1,000 repetitions for each simulation were used. The simulations were based on the original power calculations for the PACE trials, with the mean PA min/week and SD values identical to these calculations.

*Table S1: Simulation settings and results used to determine cut-points for noninferiority and superiority margins. Ctrl = Control arm, HD = high dose arm, LD = low dose arm, SD = standard deviation of the mean PA min/week for all arms, VPC = variance partition coefficient.*

| Mean PA min/week | | | SD | VPC | P(LD> HD-16.4)>0.9 & P(LD>Ctrl)>0.9 | P(LD> HD-16.4)>0.95 & P(LD>Ctrl)>0.95 | P(HD>Ctrl)>0.95 & P(HD>LD)>0.95 |
| --- | --- | --- | --- | --- | --- | --- | --- |
| Ctrl | HD | LD |  |  |  |  |  |
| 110.2 | 143 | 126.6 | 53.6 | 0.06 | 0.097 | 0.044 | 0.720 |
| 110.2 | 110.2 | 110.2 | 53.6 | 0.06 | 0.125 | 0.051 | 0.010 |
| 110.2 | 143 | 110.2 | 53.6 | 0.06 | 0.001 | 0 | 0.991 |
| 110.2 | 143 | 126.6 | 53.6 | 0.1 | 0.091 | 0.052 | 0.660 |
| 110.2 | 110.2 | 110.2 | 53.6 | 0.1 | 0.107 | 0.056 | 0.015 |
| 110.2 | 143 | 110.2 | 53.6 | 0.1 | 0 | 0 | 0.980 |

Table S1 details the simulations settings and results used to determine cut-points for non-inferiority and superiority margins. A cut-point of 0.95 for noninferiority and superiority for the observed VPC (VPC = 0.06) resulted in a maximum type 1 error rate of 0.051. A VPC higher than what was seen in the trial (VPC = 0.1) resulted in a higher type 1 error rate.
