# Supplementary material for: Bayesian adaptive designs for the optimisation of implementation strategies: a virtual re-execution of the Physically Active Children in Education (PACE) trials
Source: Implement Sci Commun. 2026 Jun 22;7:179. doi: 10.1186/s43058-026-01000-2 (PMC13548693; doi:10.1186/s43058-026-01000-2)
Supplement: Supplementary file 2 [file 43058_2026_1000_MOESM2_ESM.docx]

Table S2: MNAR sensitivity analysis. Adaptive design decisions, conclusions, and mean difference of weekly PA time administered by teachers by trial design when assuming missing follow-up PA values were 180 minutes lower than non-missing values.

|  |  |  | **Posterior probabilities** | | | | **Mean difference (95% CrI)** | |
| --- | --- | --- | --- | --- | --- | --- | --- | --- |
| **N interims** | **Adaptive design decisions** | **Conclusion** | **P(LD > HD-16.4)** | **P(LD>HD)** | **P(LD>Ctrl)** | **P(HD>Ctrl)** | **LD - HD** | **LD - Ctrl** |
| **Fixed (0)** | NA | LD optimal | 0.99 | 0.46 | >0.99 | >0.99 | -0.6 (-14.7, 13.6) | 47.0 (29.3, 64.4) |
| *Interim 1* |  |  |  |  |  |  |  |  |
| **One** | Stop early for NI | LD optimal | 0.96 | 0.59 | >0.99 | >0.99 | 2.2 (-19.3, 21.9) | 52.8 (28.1, 75.8) |
| **Two** | Stop early for NI | LD optimal | >0.99 | 0.85 | >0.99 | >0.99 | 12.7 (-11.0, 32.1) | 56.7 (29.1, 79.1) |
| **Three** | Stop early for NI | LD optimal | >0.99 | 0.85 | >0.99 | >0.99 | 13.9 (-10.7, 34.7) | 62.7 (34.3, 87.2) |
